# Comparative Analysis of Cross-Protective Immunity Among Three Geographically Distinct Isolates of *Eimeria kongi*

**DOI:** 10.3390/ani14233524

**Published:** 2024-12-05

**Authors:** Sufang Fang, Linghai Meng, Yubo Shi, Chengyu Hao, Xiaolong Gu, Fangchen Du, Ping Cui, Xinming Tang

**Affiliations:** 1 College of Animal Science and Technology, Hebei North University, Zhangjiakou 075000, China; zjkfsf@126.com (S.F.); tsgaocan@163.com (L.M.); 17360708807@163.com (C.H.); bingli2006@126.com (X.G.); dufangchen@gmail.com (F.D.); 2 Key Laboratory of Animal Biosafety Risk Prevention and Control (North), Key Laboratory of Veterinary Biological Products and Chemical Drugs of MARA, Institute of Animal Science, Chinese Academy of Agricultural Sciences, Beijing 100193, China; shiyb0123@foxmail.com

**Keywords:** rabbit coccidiosis, *Eimeria kongi*, pathogenicity, immunity, cross-protection, vaccine development

## Abstract

Coccidiosis is one of the most significant diseases affecting the rabbit industry and is caused by *Eimeria*. In a previous study, we identified a new species of *Eimeria kongi* (*E. kongi*-ZJK) from the northern region of China (Zhangjiakou, Hebei Province) and studied its pathogenicity and immunogenicity. The aim of this study was to evaluate the pathogenicity, immunogenicity, and cross-immunogenicity from different geographical isolates of *E. kongi* for vaccine development. Two geographical isolates of *E. kongi*-QD from Qingdao, Shandong Province (eastern China), and *E. kongi-*CD from Chengdu, Sichuan Province (southwestern China), were isolated and identified. The pathogenicity, immunogenicity, and cross-immunogenicity among the three geographical isolates were evaluated. The pathogenicity results showed that after infecting rabbits with doses of 1 × 10^3^ or fewer sporulated oocysts of *E. kongi*-QD and *E. kongi*-CD, the rabbits exhibited clinical symptoms but recovered quickly, indicating lower pathogenicity. Immunogenicity studies revealed that after immunizing rabbits with 1 × 10^3^ sporulated oocysts of *E. kongi*-QD and *E. kongi*-CD for 14 days, followed by challenge with 1 × 10^4^ homologous sporulated oocysts, the oocyst reduction rates in the immunized groups were 99.39% and 99.12%, respectively, compared with the non-immunized groups, demonstrating good immunogenicity. In cross-immunogenicity studies, rabbits were immunized with 1 × 10^3^ sporulated oocysts of *E. kongi*-ZJK, *E. kongi*-QD, or *E. kongi*-CD and then challenged with 1 × 10^4^ sporulated oocysts of heterologous isolate. The immunized groups showed no significant clinical symptoms, and the oocyst reduction rates ranged from 55.9% to 98.4%. *E. kongi*-CD exhibited cross-protection against *E. kongi*-ZJK and *E. kongi*-QD, making it an ideal candidate formulation for vaccine development.

## Simple Summary

We aimed to identify vaccine candidates to control rabbit coccidiosis, a major disease in the rabbit industry caused by *Eimeria* parasites. We isolated and examined three isolates of *Eimeria kongi* from different regions of China—Zhangjiakou (*E. kongi*-ZJK), Qingdao (*E. kongi*-QD), and Chengdu (*E. kongi*-CD)—and then assessed the pathogenicity, immunogenicity, and cross-protective immunity of these isolates. Results showed that *E. kongi*-QD and *E. kongi*-CD had lower pathogenicity, causing mild symptoms that resolved quickly in rabbits. Immunization with *E. kongi*-QD and *E. kongi*-CD significantly reduced oocyst output upon homologous challenge, indicating strong immunogenicity. *E. kongi*-CD also provided cross-protection against both *E. kongi*-ZJK and *E. kongi*-QD, suggesting it could be a promising candidate for coccidiosis vaccine development.

## 1. Introduction

The rabbit industry is a vital component of the global livestock sector, particularly producing rabbit meat, which is highly regarded for its nutritional benefits, including its low in fat and rich in protein [1]. However, infectious diseases remain a major bottleneck in the growth and sustainability of this industry [2]. Among these, coccidiosis, caused by parasites of the genus *Eimeria*, is one of the most prevalent and economically damaging diseases affecting rabbits worldwide [3,4]. Currently, there are 11 recognized species of *Eimeria* that infect rabbits, along with one newly identified species, *Eimeria kongi* (the naming is in honor of the renowned parasitologist, Professor Kong Fanyao from China Agricultural University), discovered in China [5,6]. Previous research has shown that the main biological characteristics of the *E. kongi*-ZJK isolate (isolated from Zhangjiakou City, Hebei Province, northern China) were systematically identified using morphological and molecular biological techniques, indicating that it is a new species of *Eimeria* [5,7]. The parasite mainly infects the epithelial cells of the jejunum and ileum of rabbits and undergoes four generations of schizogony, with a prepatent period of 132 h. The size of the sporulated oocyst is 32.6–41.2 μm × 20.9–25.5 μm, containing a sporocyst residual body but no oocyst residual body. The shortest sporulation time for the oocysts is approximately 40 h [5]. Young rabbits infected with this *Eimeria* isolate exhibited diarrhea, growth suppression, and, in severe cases, death, indicating moderate pathogenicity. After inoculation with a certain dose of sporulated oocysts, homologous challenge tests demonstrated good immunogenicity [5].

The current control of rabbit coccidiosis mainly relies on anticoccidial drugs [8,9]. However, with the emergence of drug-resistant strains, the effectiveness of these drugs is no longer sufficient to meet the needs of large-scale rabbit farming [9,10]. Another strategy for controlling coccidiosis is the development of vaccines based on live oocysts, which have already been successfully applied and promoted for the control of chicken coccidiosis [11,12]. Rabbit coccidiosis vaccines have a long research history, but few products have been commercialized [12,13]. Several factors limit the commercialization of rabbit coccidiosis vaccines, including but not limited to the following: (1) Diverse *Eimeria* species: There are numerous species of *Eimeria* in rabbits, with new species continuously being reported [5,14]. Different species lack cross-immunity, and even between different geographical isolates/strains of the same species, cross-immunity varies [11,15,16]. This phenomenon is especially prominent in *E. maxima* in chicken [17]. Therefore, the development of rabbit coccidiosis vaccines requires a multivalent approach tailored to the prevalent isolates/strains, complicating vaccine development. (2) High production requirements: Whether derived from wild strains or attenuated strains, the production of live vaccines requires the use of rabbits, with oocysts extracted from feces or intestinal contents for sporulation [11]. However, compared to chicken coccidia, the consistent control of sporulation rates between batches of rabbit coccidia oocysts is challenging, which is critical for ensuring the safety of live vaccines [18]. (3) Varying rabbit farming scales and practices: Different countries and regions have significant differences in rabbit farming scale and methods, and the level of intensification is not comparable to poultry farming [2]. This variation makes it difficult to ensure the effectiveness of live vaccines in farms with poor management practices. (4) Risk of coccidiosis outbreaks: Like other live vaccines, improper management of rabbit coccidiosis vaccines carries the risk of causing outbreaks of the disease [19].

To further understand the prevalence of the newly identified *E. kongi* in China, we isolated and identified *E. kongi* from major rabbit farming regions, specifically Qingdao in the eastern region of Shandong Province and Chengdu in the southwestern region of Sichuan Province. These isolates were named *E. kongi*-QD (Qingdao) and *E. kongi*-CD (Chengdu). We systematically studied the pathogenicity and immunogenicity of the different geographical isolates. On this basis, we investigated the cross-protective immunity among the three geographical isolates to support the development of a vaccine strain for *E. kongi*.

## 2. Materials and Methods

### 2.1. Ethics Statement

All animal experiments were performed in strict accordance with the Animal Welfare Ethics Committee of Hebei North University guidelines (HBNU20230631118) and followed the International Guiding Principles for Biomedical Research Involving Animals. Experiments were approved by the Hebei Administration Committee of Laboratory Animals.

### 2.2. Animals and Parasites

New Zealand White rabbits aged 35 days with similar body weights and in good health (showing no signs of lethargy, diarrhea, or other abnormal clinical symptoms) were selected for the experiment. Rabbit feces were examined for the presence of oocysts using the saturated saline flotation method with a McMaster counting chamber. Rabbits were fed a diet and provided drinking water without any anticoccidial drugs added. Daily fecal oocyst checks were performed, and the rabbits were housed in isolation units until they reached 45 days of age, ensuring no coccidia infection before the experiment [11,20].

*Eimeria kongi*-ZJK: isolated from the rabbit farm in Saibei, Zhangjiakou, Hebei Province [5]; *E. kongi*-QD: isolated from the California rabbit farm in Qingdao, Shandong Province; *E. kongi*-CD: isolated from the Jianxiang Rex rabbit farm in Chengdu, Sichuan Province. Sporulated oocysts from the above three geographical isolates were preserved in 2.5% potassium dichromate solution and stored at 4 °C in the Animal Parasitology Laboratory of Hebei North University. Prior to the experiments, the oocysts were purified using sodium hypochlorite [7] and revitalized every six months.

### 2.3. Analysis of the ITS-1 and cox1 Sequences of E. kongi-QD and E. kongi-CD

*E. kongi*-QD and *E. kongi*-CD sporulated oocysts (1 × 10^3^) were inoculated into coccidia-free rabbits for propagation. Oocysts were collected and purified, and genomic DNA was extracted from the oocysts, as described previously [7]. The ITS-1 (ITS1-F: 5′-AAGTTGCGTAAATAGAGCCC-3′; ITS1-R: 5′-CAAGACATCCATTGCTGAAA-3′) and the conserved region of cox1 (cox1-F: 5′-GTTTGGTTCAGGTGTTGGTTGGAC-3′; cox1-R: 5′-ATCCAATAACCGCACCAAGAGATA-3′) sequences of *Eimeria* species were amplified using *Eimeria*-specific primers with predicted products of 413 bp and 809 bp in size, respectively [21]. The PCR conditions were set as follows: initial denaturation at 94 °C for 4 min, followed by 30 cycles of denaturation at 94 °C for 30 s, annealing at 56 °C for 15 s, and extension at 72 °C for 45 s, with a final extension at 72 °C for 10 min. The total reaction volume was 25 μL. The amplified products were sequenced by Beijing Ruibo Xingke Biotechnology Co., Ltd. (Beijing, China). PCR amplification was performed using ddH_2_O as the template as a negative control. The obtained ITS-1 and cox1 sequences were then compared with *Eimeria* ITS-1 and cox1 sequences in GenBank to construct a phylogenetic tree.

### 2.4. Pathogenicity and Immunogenicity of E. kongi-QD and E. kongi-CD

Sixty-six 45-day-old coccidia-free rabbits were randomly divided into 11 groups. The rabbits were inoculated with 1 × 10^2^, 1 × 10^3^, 1 × 10^4^, and 5 × 10^4^ sporulated oocysts per rabbit of *E. kongi*-QD or *E. kongi*-CD, respectively. Rabbits inoculated with the same volume of PBS were used as unimmunized challenged control groups (UCCs), and the experiment also included an unimmunized unchallenged control group (UUC). The immunized rabbits were challenged at 14 days after immunization with 1 × 10^4^ homologous sporulated oocysts per rabbit. After inoculation/immunization and challenge infection, the rabbits were closely monitored for changes in behavior, water consumption, feed intake, and fecal color and consistency. Each group’s body weight was recorded every seven days, and the average daily weight gain was calculated both after inoculation and challenge. Fecal samples were collected and weighed 7–11 days post-inoculation and post-challenge to assess the number of oocysts shedding.

### 2.5. Cross-Immunogenicity Analysis of the Three Geographical Isolates

Forty-five-day-old rabbits free of coccidia were divided into 10 groups, with 6 rabbits in each group. The ZJK-QD and ZJK-CD groups were orally immunized with 1 × 10^3^ *E. kongi*-ZJK sporulated oocysts each. The QD-ZJK and QD-CD groups were orally immunized with 1 × 10^3^ *E. kongi*-QD sporulated oocysts per rabbit, and the CD-QD and CD-ZJK groups were orally immunized with 1 × 10^3^ *E. kongi*-CD sporulated oocysts. The UCC-ZJK, UCC-QD, and UCC-CD groups were the unimmunized challenged control groups (UCCs), and after 14 days of immunization, each rabbit in the respective groups was challenged orally with 1 × 10^4^ sporulated oocysts of *E. kongi*-ZJK, *E. kongi*-QD, or *E. kongi*-CD. The experiment included a blank control group (UUC, unimmunized unchallenged control group). Details of the immunized strains and challenge doses are shown in Table 1. Following immunization, the rabbits’ water and food intake, general behavior, stool shape, and stool color were observed and recorded daily. The trial period lasted 28 days, during which the rabbits’ weight was measured every 7 days. Fecal samples were collected from days 7 to 11 after immunization and from days 7 to 11 after challenge to calculate the number of oocysts shed. The cross-protective immunity among the different geographical isolates of *E. kongi* was determined based on changes in clinical symptoms, weight, oocyst shedding, and weight gain.

### 2.6. Statistical Analysis

The data were analyzed using SPSS 22.0 statistical software, and results for each group are presented as mean ± standard deviation (SD). Group differences were evaluated using Duncan’s Multiple Range Test following ANOVA. Statistical significance was defined as follows: *p* ≤ 0.05 was considered significant, *p* < 0.01 was regarded as highly significant, and *p* > 0.05 indicated no significant difference.

## 3. Results

### 3.1. The Systematic Identification of E. kongi-QD and E. kongi-CD

#### 3.1.1. Morphological Analysis of Sporulated Oocysts

**Figure 1:**
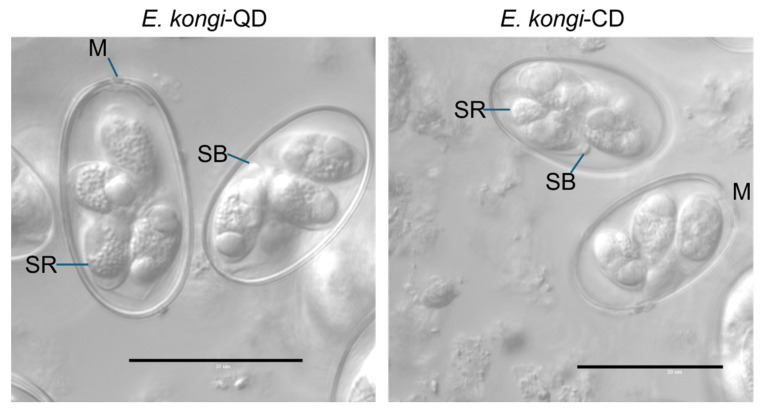
Sporulated oocyst of *E. kongi*-QD and *E. kongi*-CD. SR, sporocyst residuum; M, micropyle; SB, Stieda body; scale bar = 30 μm.

The two isolates of *Eimeria* oocysts we isolated exhibited the following characteristics: the oocysts were ellipsoidal to slightly ovoidal in shape, with a double-layered wall that was thick and smooth. The micropyle (M) was clearly visible, and the sporocysts were ellipsoidal to elongate, containing both a Stieda body (SB) and sporocyst residuum (SR) but no oocyst residuum. The shape index of the oocysts was approximately 1.6 (Figure 1). These features are consistent with the morphological characteristics of *E. kongi* [5].

#### 3.1.2. Genetic Phylogenetic Analysis and Identification Based on ITS-1 Sequences

**Figure 2:**
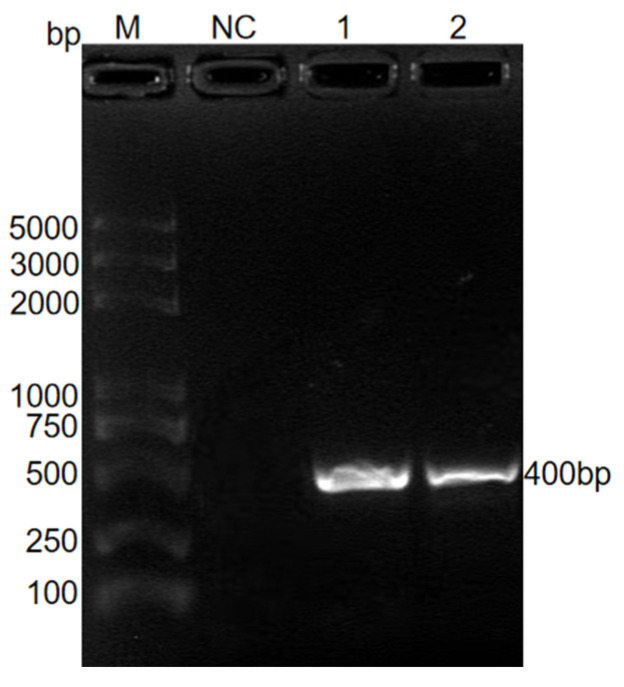
PCR product of ITS-1 sequence of *E. kongi*-QD and *E. kongi*-CD. M, DNA marker; NC, negative control; Lane 1, *E. kongi*-QD; Lane 2, *E. kongi*-CD.

Using ITS-1 conserved primers, amplification of *E. kongi*-QD and *E. kongi*-CD resulted in clear target bands, which matched the expected 413 bp in size (Figure 2). The target bands were excised, cloned, and sequenced, and the ITS1 sequence similarity between *E. kongi*-QD and *E. kongi*-ZJK was 99%, while that between *E. kongi*-CD and *E. kongi*-ZJK was 98%.

**Figure 3:**
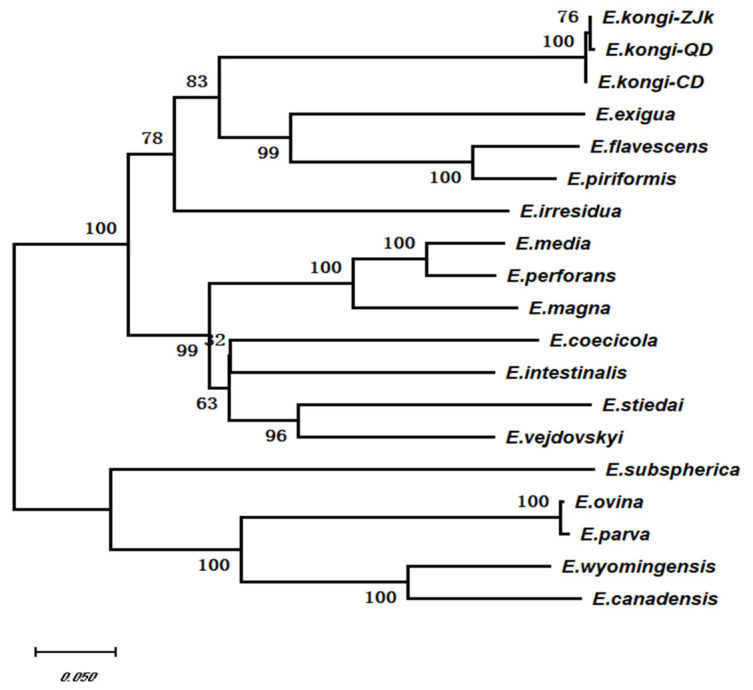
Phylogenetic analysis *E. kongi*-QD and *E. kongi*-CD based on ITS-1 sequence.

Using the ITS-1 sequences from 11 species of rabbit coccidia available in GenBank, along with the ITS-1 sequences of *E. kongi*-ZJK, *E. kongi*-QD, and *E. kongi*-CD as the in-group, and the ITS-1 sequences from coccidia parasitizing cattle and sheep as the out-group, a total of 16 sequences were subjected to multiple sequence alignment analysis (Table 2). Using the MEGA X software (Version 10.2.6), phylogenetic analysis of the ITS-1 sequences was performed using the Neighbor-Joining (NJ) method, and a phylogenetic tree was constructed (Figure 3). The results showed that *Eimeria* species parasitizing cattle and sheep form separate monophyletic groups from those parasitizing rabbits. Based on the presence or absence of residual bodies, rabbit *Eimeria* species were divided into two sister lineages: a lineage without residual bodies and a lineage with residual bodies. *E. kongi*-QD and *E. kongi*-CD clustered together with *E. kongi*-ZJK in a small branch and grouped within the no-residual-body lineage (including *E. kongi*-ZJK, *E. kongi*-QD, *E. kongi*-CD, *E. exigua*, *E. piriformis*, *E. flavescens*, and *E. irresidua*), while the remaining species were grouped in the other lineage (Figure 3).

#### 3.1.3. Genetic Phylogenetic Analysis and Identification Based on cox1 Sequences

**Figure 4:**
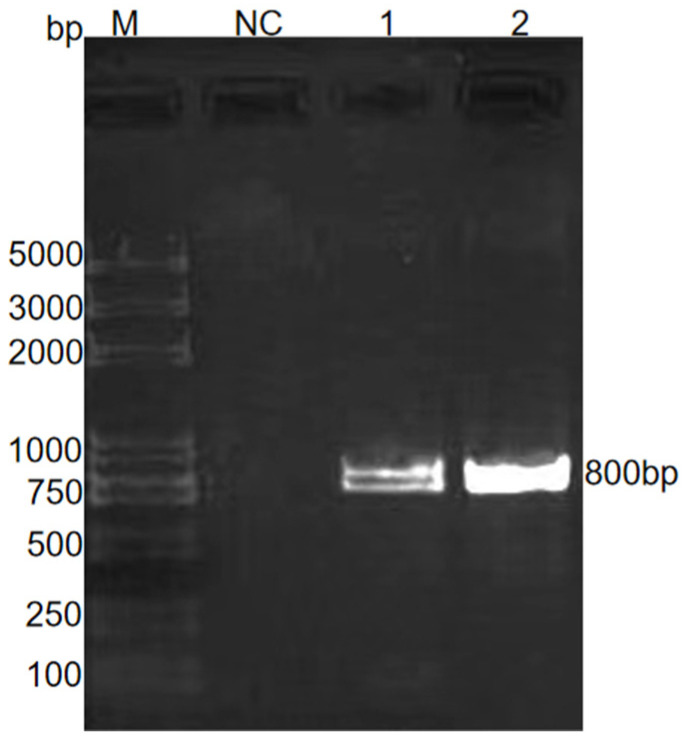
PCR product of cox1 sequence of *E. kongi*-QD and *E. kongi*-CD. M, DNA marker; NC, negative control; Lane 1, *E. kongi*-QD; Lane 2, *E. kongi*-CD.

Amplification of *E. kongi*-QD and *E. kongi*-CD was performed using conserved cox1 primers. Clear target bands were observed, which matched the expected 809 bp in size (Figure 4). The conserved region of the cox1 genes of *E. kongi*-QD and *E. kongi*-CD showed 100% similarity with the cox1 gene of *E. kongi*-ZJK.

**Figure 5:**
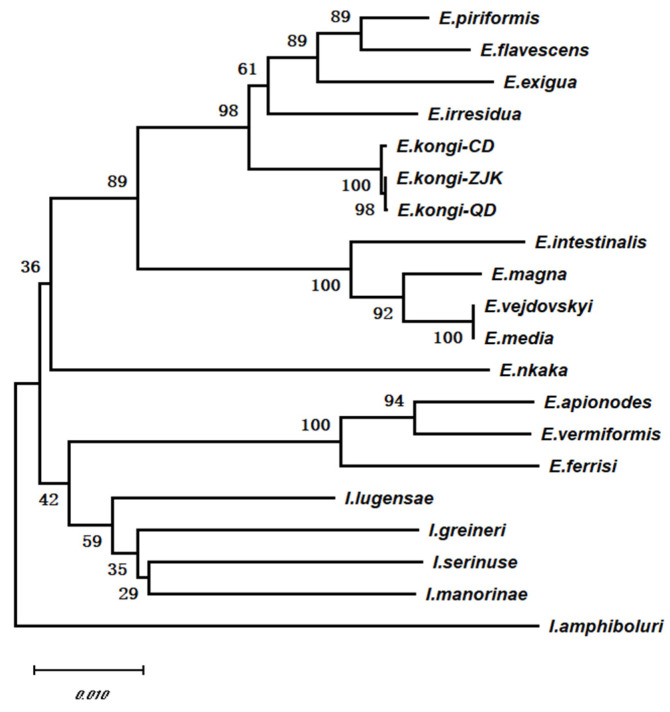
Phylogenetic analysis *E. kongi*-QD and *E. kongi*-CD based on cox1 sequence.

Using the cox1 genes of eight previously submitted rabbit *Eimeria* species and other coccidian species (Table 3), a phylogenetic tree was constructed (Figure 5). The results showed that *Eimeria* and *Isospora* each formed their own distinct monophyletic groups. The rabbit *Eimeria* monophyletic group was divided into two sister clades based on the presence or absence of an oocyst residual body. *E. kongi*-QD and *E. kongi*-CD clustered with *E. kongi*-ZJK in the clade without a residual body (*E. kongi*-ZJK, *E. kongi*-QD, *E. kongi*-CD, *E. exigua*, *E. piriformis*, *E. flavescens*, *E. irresidua*), while other rabbit *Eimeria* species with a residual body grouped in a different clade (Figure 5).

### 3.2. Comparison of Pathogenicity and Immunogenicity of E. kongi-QD and E. kongi-CD

#### 3.2.1. Clinical Symptoms

On the fourth day post-inoculation, rabbits in the 1 × 10^2^ dose group exhibited reduced appetite and soft stool, but no diarrhea was observed. In the 1 × 10^3^ dose group, rabbits displayed lethargy, reduced appetite, and mild diarrhea. In the 1 × 10^4^ dose group, rabbits showed depression, reduced appetite, and diarrhea, with 50% of the rabbits in the *E. kongi*-QD group developing diarrhea and 16.67% in the *E. kongi*-CD group. In the 5 × 10^4^ dose group, all rabbits exhibited reduced appetite, severe lethargy, and diarrhea, and by the seventh day post-inoculation, one rabbit in the *E. kongi*-QD group died due to *E. kongi* infection. By the eighth to ninth day, the rabbits in the 1 × 10^2^ and 1 × 10^3^ groups recovered, and by the twelfth day, all clinical symptoms disappeared.

On the fourth day post-challenge, rabbits in the non-immunized challenge control group exhibited reduced food intake and diarrhea, returning to normal by the twelfth day. In the 1 × 10^2^ group, rabbits showed reduced food intake but no diarrhea, while those in the 1 × 10^3^, 1 × 10^4^, and 5 × 10^4^ groups showed normal food intake, normal behavior, and no diarrhea. On the fifth to sixth day post-challenge, rabbits in the 5 × 10^4^ group showed reduced appetite and diarrhea, with diarrhea rates of 50% in the *E. kongi*-QD group and 33.33% in the *E. kongi*-CD group. By the eighth day post-challenge, all clinical symptoms in the challenged groups had disappeared.

#### 3.2.2. Oocyst Shedding

**Figure 6:**
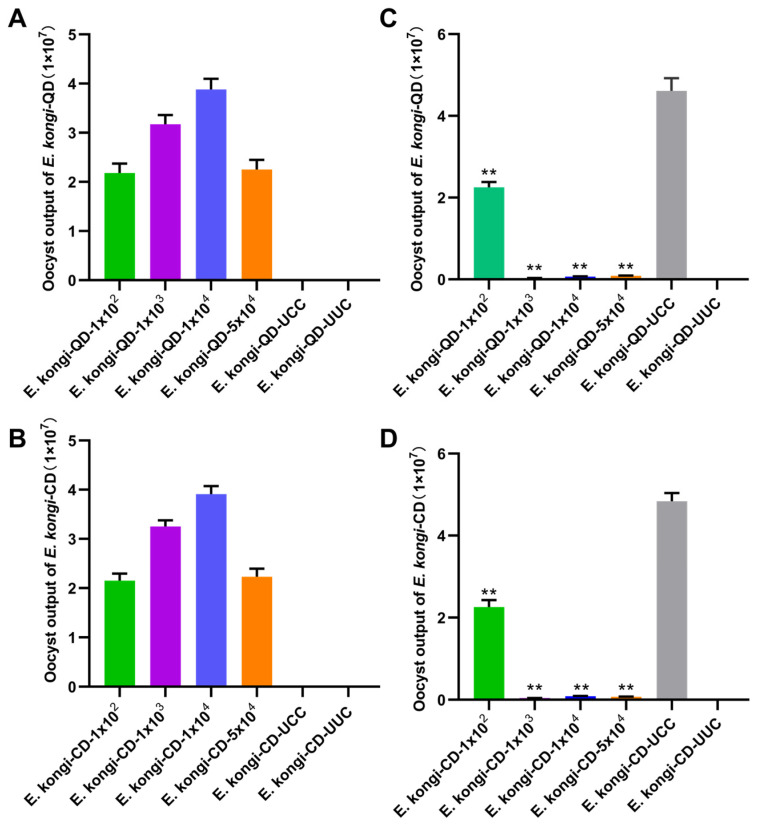
Oocyst shedding following immunization and challenge infection with *E. kongi*-QD and *E. kongi*-CD. (**A**) Oocyst shedding from days 7 to 11 after inoculation with different doses of *E. kongi*-QD. (**B**) Oocyst shedding from days 7 to 11 after inoculation with different doses of *E. kongi*-CD. (**C**) Oocyst shedding from days 7 to 11 after high-dose challenge infection with the *E. kongi*-QD following immunization with the same strain. (**D**) Oocyst shedding from days 7 to 11 after high-dose challenge infection with the *E. kongi*-CD following immunization with the same strain. ** Indicates a significant difference compared with the unimmunized group (UCC).

Oocyst shedding began at 6.5 days post-inoculation in all infected groups, reaching a peak on day 10, and by day 14, only minimal oocyst shedding was observed. The total oocyst counts from days 7 to 11 in the *E. kongi*-QD inoculated groups were 2.18 × 10^7^, 3.26 × 10^7^, 3.88 × 10^7^, and 2.25 × 10^7^ oocysts, respectively (Figure 6A). For the *E. kongi*-CD-inoculated groups, the total oocyst counts during the same period were 2.15 × 10^7^, 3.25 × 10^7^, 3.91 × 10^7^, and 2.23 × 10^7^ oocysts, respectively (Figure 6B). These results indicate that the number of oocyst shedding significantly increased with the increase in the infection dose. However, when the infection dose was too high (for example, 5 × 10^4^ oocysts/rabbit), the oocyst shedding actually decreased, showing a crowding effect.

Following the challenge infection, the total oocyst counts from days 7 to 11 post-challenge significantly decreased in all immunized groups compared with the unimmunized challenged control group (UCC) (*p* < 0.01) (Figure 6C,D). The oocyst reduction rates in the *E. kongi*-QD groups were 51.17%, 99.39%, 98.58%, and 98.10% for the 1 × 10^2^, 1 × 10^3^, 1 × 10^4^, and 5 × 10^4^ doses of immunized groups, respectively. In the *E. kongi*-CD groups, the oocyst reduction rates were 53.27%, 99.12%, 98.23%, and 98.48% for the same dose of immunized groups. These results indicate that a single low-dose immunization (10^3^ oocysts/rabbit or more) can provide good immune protection against subsequent infections with homologous strains.

#### 3.2.3. Body Weight Gain

**Figure 7:**
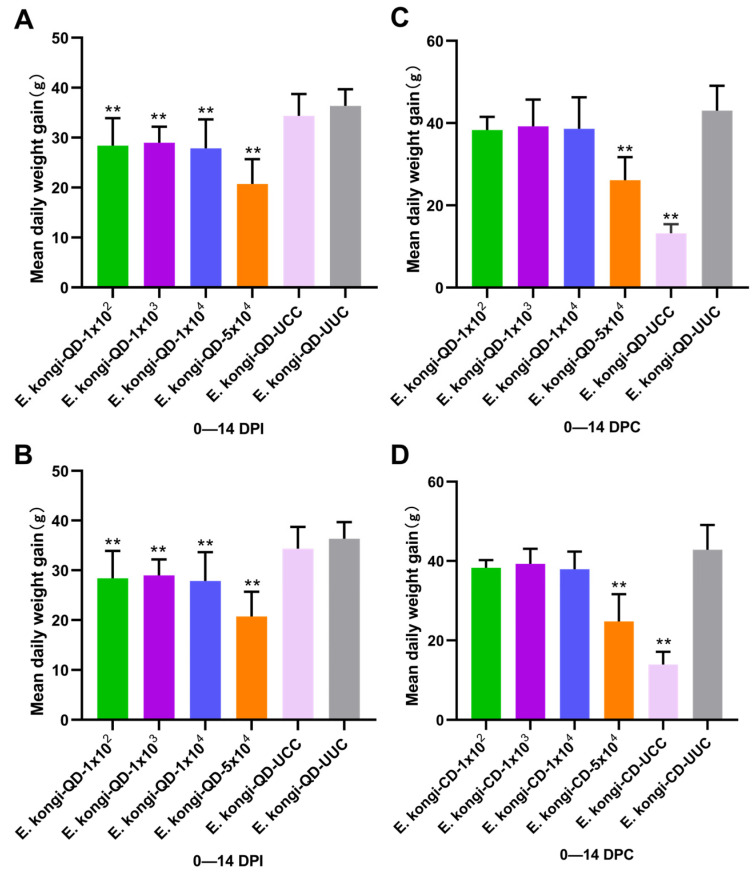
Mean body weight gain following immunization and challenge infection with *E. kongi*-QD and *E. kongi*-CD. (**A**) Mean body weight gain from days 0 to 14 after inoculation with different doses of *E. kongi*-QD. (**B**) Mean body weight gain from days 0 to 14 after inoculation with different doses of *E. kongi*-CD. (**C**) Mean body weight gain from days 0 to 14 after high-dose challenge infection with the *E. kongi*-QD following immunization with the same strain. (**D**) Mean body weight gain from days 0 to 14 after high-dose challenge infection with the *E. kongi*-CD following immunization with the same strain. ** Indicates a significant difference compared to the unimmunized group (UCC).

At 14 days post-inoculation with *E. kongi*-QD or *E. kongi*-CD, the average daily weight gain of rabbits in all inoculated groups decreased, with the reduction in weight gain correlating with the increase in inoculation dose. The differences in weight gain between the inoculated groups and the unimmunized unchallenged control group (UUC) were highly significant (*p* < 0.01) (Figure 7A,B).

At 14 days post-challenge, the average daily weight gain in the 5 × 10^4^ group was significantly different from that of the UUC group (*p* < 0.01), while the differences in weight gain between other inoculated groups and the UUC group were not significant (*p* > 0.05) (Figure 7C,D).

### 3.3. Cross-Immunogenicity of the E. kongi Three Geographical Isolates

#### 3.3.1. Clinical Symptoms

After the challenge infection, the rabbits in the immunized groups exhibited good mental condition and appetite, with normal stool appearance compared with UUC. In contrast, rabbits in the unimmunized challenged groups displayed clinical symptoms, including reduced appetite, lethargy, and diarrhea on the fourth day post-challenge. The diarrhea rate reached 50% in the UCC-ZJK and UCC-QD groups and 33.33% in the UCC-CD group. By the eighth day post-challenge, these symptoms began to subside, and by the eleventh day, all rabbits in the unimmunized challenged groups had returned to normal.

#### 3.3.2. Oocyst Shedding

**Figure 8:**
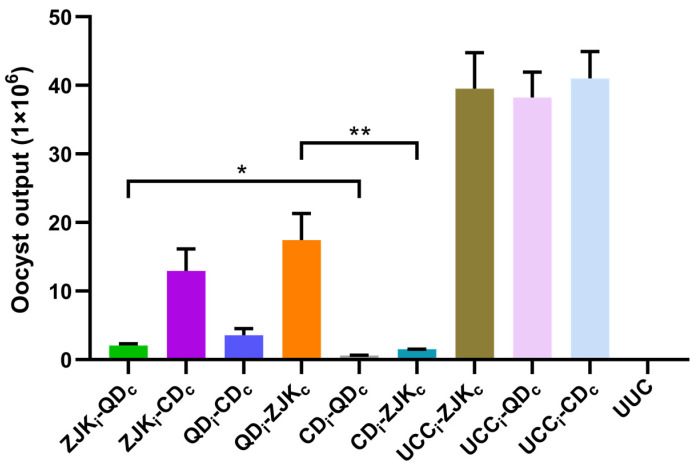
The oocyst shedding after heterologous challenge infection following immunization with different geographical isolates of *E. kongi.* The subscript “i” represents the immunizing isolate of *E. kongi*, and the subscript “c” represents the challenge isolate of *E. kongi*. * Indicates there is a significant difference between the two groups; ** Indicates there is a highly significant difference between the two groups.

Between days 7 and 11 post-challenge, all rabbits immunized with different isolates exhibited a significant reduction in total oocyst shedding compared with each unimmunized control (*p* < 0.01) (Figure 8). The inhibition rates of oocyst shedding were calculated, showing that *E. kongi*-CD effectively suppressed oocyst shedding after the challenge with *E. kongi*-ZJK and *E. kongi*-QD, with inhibition rates of 98.4% and 96.2%, respectively.

#### 3.3.3. Body Weight Gain

**Figure 9:**
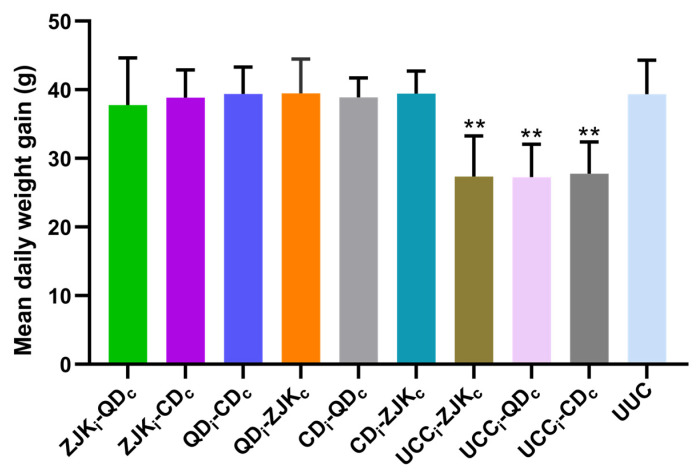
The mean body weight gain after heterologous challenge infection following immunization with different geographical isolates of *E. kongi.* The subscript “i” represents the immunizing isolate of *E. kongi*, and the subscript “c” represents the challenge isolate of *E. kongi*. ** Indicates a significant difference compared to the unchallenged group (UUC).

Fourteen days after the challenge, there was no significant difference in the average daily weight gain between the immunized challenged groups and the unimmunized-unchallenged group (UUC) during the 0–14-day period (*p* > 0.05). However, the unimmunized challenged groups showed a significantly lower average daily weight gain compared with the UUC group (*p* < 0.01) (Figure 9).

## 4. Discussion

In this study, we systematically identified geographic strains of *E. kongi* isolated from different regions of China, which partially reflects the prevalence of *E. kongi* in the country [22]. Furthermore, we analyzed the pathogenicity, immunogenicity, and cross-immunogenicity of these strains, identifying strains with low pathogenicity, good immunogenicity, and strong cross-protective immunity among different geographical strains. These strains provide valuable seed stock for the development of live vaccines against coccidiosis.

Based on the morphological structure of sporulated oocysts, prepatent period, and sporulation time, *E. kongi*-QD and *E. kongi*-CD were isolated. To accurately identify these strains, two molecular genetic markers were used to construct a phylogenetic tree for molecular identification. A phylogenetic tree based on a single genetic marker represents only the evolutionary history of that specific marker, which may not reflect the actual phylogeny of *Eimeria*. It is necessary to ensure consistency across multiple genetic markers, ideally including at least one nuclear gene or sequence and one cytoplasmic gene, such as mitochondrial or plastid genes [23]. Therefore, in this study, the ITS-1 sequence and cox1 gene were selected as molecular genetic markers to construct the phylogenetic tree, confirming that *E. kongi*-QD and *E. kongi*-CD are two geographical strains of *E. kongi*.

Different geographical strains of rabbit coccidia exhibit varying virulence levels. For example, the lethal dose (LD50) of *E. intestinalis* isolated from China is 5 × 10⁵ sporulated oocysts, while the LD50 of French and Czechoslovakian strains is approximately 6 × 10^3^ sporulated oocysts, indicating significantly lower virulence in the Chinese strain [24]. Historically, *E. magna* has been considered moderately pathogenic, yet a strain isolated from Hebei Province showed high pathogenicity, highlighting the importance of clarifying virulence among different geographical strains [12,13,25,26].

To assess the virulence of *E. kongi*-QD and *E. kongi*-CD, rabbits were inoculated with four doses of sporulated oocysts (1 × 10^2^, 1 × 10^3^, 1 × 10^4^, 5 × 10^4^), and their clinical symptoms were observed. Results indicated that even the lowest dose of 1 × 10^2^ oocysts caused decreased appetite in rabbits, while the 1 × 10^3^ dose induced diarrhea. At the 5 × 10^4^ dose, diarrhea rates significantly increased, and one rabbit died, indicating that both *E. kongi*-QD and *E. kongi*-CD have moderate pathogenicity [5].

Immunogenicity was evaluated by inoculating rabbits with 1 × 10^3^ sporulated oocysts of *E. kongi*-QD or *E. kongi*-CD, followed by a challenge with 1 × 10^4^ homologous oocysts 14 days later. There was no significant difference in weight gain between the immunized and control groups (*p* < 0.05), and the oocyst inhibition rates were 99.39% and 99.12%, respectively, demonstrating good immunogenicity. 

*Eimeria* species exhibit complex antigenic compositions, with antigenic differences between species and even among strains of the same species, as well as between different developmental stages [18]. Long (1979) reported that immunizing animals with mixed oocysts of different *E. maxima* strains conferred good cross-protection, even with small doses [27]. Using weight gain and lesion scores to analyze cross-protection among five *E. maxima* strains, the results showed that three strains provided cross-immunity [28]. Another study found that the host’s genetic background influenced cross-protection in *E. maxima* [29].

There has been limited research into cross-immunity in rabbit coccidia. Our research team isolated three geographical strains: *E. kongi*-ZJK from Zhangjiakou, *E. kongi*-QD from Qingdao, and *E. kongi*-CD from Chengdu. These strains were isolated from geographically distant regions and different rabbit breeds. Using average daily weight gain and oocyst inhibition rates to measure cross-protective immunity among the three strains, we found that they all provided good immunity. *E. kongi*-CD demonstrated higher cross-protection against *E. kongi*-ZJK and *E. kongi*-QD, making it a promising candidate strain for future vaccine development.

## 5. Conclusions

This study provides a comprehensive analysis of the pathogenicity, immunogenicity, and cross-protective immunity of three geographically distinct isolates of *E. kongi* from different regions in China. The findings indicate that *E. kongi*-CD demonstrates both low pathogenicity and strong cross-protective immunity, making it a promising candidate for vaccine development. The results also highlight the significant variation in virulence and immunogenicity among different geographical isolates, emphasizing the necessity for multivalent vaccine formulations tailored to regional strains. This work contributes to the understanding of cross-protective immunity in rabbit coccidia and lays the foundation for developing effective live vaccines to combat this economically impactful disease in rabbit farming. Future research should focus on optimizing vaccine formulations and further evaluating the genetic and antigenic diversity of *Eimeria* species.

## Figures and Tables

**Table 1:** Cross-immunogenicity experiment grouping of different geographical isolates of *E. kongi*.

Group	Immunization Isolate	Immunization Dose	Challenge Isolate	Challenge Dose
ZJK-QD	*E. kongi*-ZJK	1 × 10^3^	*E. kongi-*QD	1 × 10^4^
ZJK-CD	*E. kongi*-ZJK	1 × 10^3^	*E. kongi-*CD	1 × 10^4^
UCC-ZJK	—	—	*E. kongi*-ZJK	1 × 10^4^
QD-ZJK	*E. kongi*-QD	1 × 10^3^	*E. kongi-*CD	1 × 10^4^
QD-CD	*E. kongi*-QD	1 × 10^3^	*E. kongi*-ZJK	1 × 10^4^
UCC-QD	—	—	*E. kongi-*QD	1 × 10^4^
CD-QD	*E. kongi*-CD	1 × 10^3^	*E. kongi-*QD	1 × 10^4^
CD-ZJK	*E. kongi*-CD	1 × 10^3^	*E. kongi*-ZJK	1 × 10^4^
UCC-CD	—	—	*E. kongi-*CD	1 × 10^4^
UUC	—	—	—	

**Table 2:** GenBank accession numbers for the 16 ITS-1 of *Eimeria* species in this study.

Species	Accession Number	Host
*E. coecicola*	HM768881	rabbit
*E. exigue*	HM768882	rabbit
*E. flavescens*	HM768883	rabbit
*E. intestinalis*	HM768884	rabbit
*E. irresidua*	HM768885	rabbit
*E. magna*	HM768886	rabbit
*E. media*	HM768887	rabbit
*E. perforans*	HM768888	rabbit
*E. piriformis*	HM768889	rabbit
*E. stiedai*	HM768890	rabbit
*E. vejdovskyi*	HM768891	rabbit
*E. ovina*	MG836234	sheep
*E. parva*	MG836233	sheep
*E. subsphericas*	AB769815	cattle
*E. wyomingensis*	AB769823	cattle
*E. canadensis*	AB769783	cattle

**Table 3:** GenBank accession numbers of *Eimeria* and *Isospora* species in this study.

Species	Accession Number	Species	Accession Number
*E. exigue*	HM768882	*E. ferrisi*	MH777562
*E. flavescens*	HM768883	*E. apionodes*	KU215487
*E. intestinalis*	HM768884	*E. vermiformis*	MH777516
*E. irresidua*	HM768885	*I. amphiboluri*	KR108297
*E. magna*	HM768886	*I. lugensae*	MW303519
*E. media*	HM768887	*I. greineri*	KR108298
*E. piriformis*	HM768889	*I. manorinae*	KX276861
*E. vejdovskyi*	HM768891	*I. serinuse*	KX276860

## Data Availability

The genome sequences are available in the NCBI, and Table 1 and Table 2 have listed all strains’ accession numbers.
